# Supplementary material for: Deep generative model super-resolves spatially correlated multiregional climate data
Source: Sci Rep. 2023 Apr 25;13:5992. doi: 10.1038/s41598-023-32947-0 (PMC10130044; doi:10.1038/s41598-023-32947-0)
Supplement: Supplementary file 1 — Supplementary Information. [file 41598_2023_32947_MOESM1_ESM.pdf]

Deep generative model super-resolves spatially correlated multiregional climate data— supplemental material

Norihiro Oyama,[1] Noriko N. Ishizaki,[2] Satoshi Koide,[1] and Hiroaki Yoshida[1]

[1] *Toyota Central R&D Labs, Inc., Bunkyo-ku, Tokyo 112-0004, Japan*

[2] *Center for Climate Change Adaptation, National Institute for Environmental Studies, Tsukuba 305-8506, Japan.*

I. TECHNICAL DETAILS

A. Architectures of networks

The network architecture is depicted in Fig. S1. In this figure, *Conv* stands for the convolution layer, *Dense* represents the fully connected layer, and *ReLU* means Rectified Linear Unit. The numbers and alphabets above the Convs represent the hyperparameters: the numbers after k, n, and s are the kernel size, the number of kernels, and the stride, respectively. The number of kernels in the pixel shuffle layers R is determined by the scale factor r as $R = 64 \times r^2$. There can be multiple pixel shuffle layers if the scale factor can be factorizable (in that case, each pixel shuffle layer has a factorized prime number value as the scale factor, e.g., 2 and 5 if the scale factor is 10). All Leaky ReLU layers in the discriminator network employ the slope of 0.2 for negative inputs.

Following the conventional architectural design used in SRGAN for image processing, our generator network comprises of a pre-processing stage consisting of a convolution operation with ReLU activation, followed by 16 residual blocks equipped with skip connections, and pixel-shuffling units, culminating with a final convolution layer. It is widely acknowledged that the use of residual blocks enables us to increase the depth of our network without encountering issues such as gradient loss, gradient explosion, or degradation. Additionally, the implementation of pixel-shuffling units is preferred over simple upsampling techniques like deconvolution due to their ability to eliminate undesired checkerboard pattern artifacts.

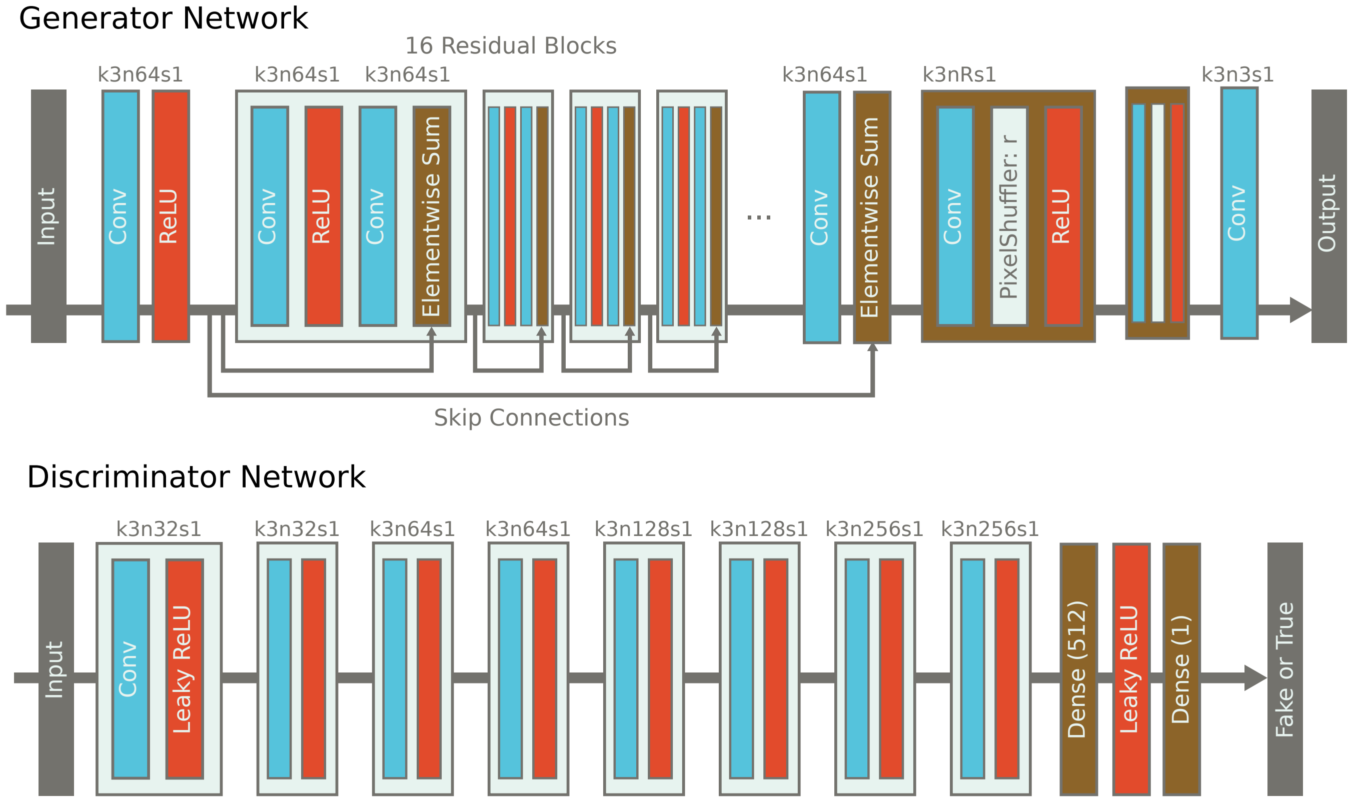

FIG. S1. Schematic pictures of the architectures of the generator and discriminator networks.

B. Learning protocol

In this section, we explain the details of the learning protocol. The protocol basically obeys ref. [1]. The fifty-fold downscaling is composed of two stages: the low-resolution to medium-resolution (LR-to-MR) and the medium-resolution to high-resolution (MR-to-HR) downscaling stages. The scale factors for these two stages are ten- and fivefold, respectively, and a fifty-fold scaling factor is achieved overall. The MR ground truths for the learning are generated by coarse-graining the HR image (average pooling with the kernel of size 5 and the stride is set to 5). We also introduced pretraining for the Generator network as usual. In total, there are four stages of learning: pretraining and GAN training for both LR-to-MR and MR-to-HR downscaling. We summarize the number of epochs and the batch size for each training stage in Table S1. The same value of the initial learning rate, $r_l = 10^{-4}$, is employed for all learning stages, and the learning rate is multiplied by 0.99 every epoch. Under this exponentially decreasing-learning-rate protocol, the learning rate becomes approximately one tenth every 230 steps. Comparing the loss values for training and validation data during the training process, we confirmed that these combinations of the epoch numbers and the learning rate prevent the system from overtraining. Other hyperparameters, such as the ones for Adam algorithm, are set to standard values used in most studies.

TABLE S1. Epoch number and batch size for each training state

	LR to MR		MR to HR	
	pre-training	GAN	pre-training	GAN
Epoch number	200	500	200	100
Batch size	100	100	50	5

Regarding the loss function, again, we obeyed ref. [1]. We employed the simple MSE loss $\mathcal{L}_{\mathrm{MSE}}$ for the pretraining regardless of the resolution stages. For the loss of the Generator network in the GAN part $\mathcal{L}_{\mathrm{G}}$, we use the linear combination of $\mathcal{L}_{\mathrm{MSE}}$ and the adversarial loss $\mathcal{L}_{\mathrm{Adv}}$:

$$\mathcal{L}_{\mathrm{G}} = \mathcal{L}_{\mathrm{MSE}} + \alpha \mathcal{L}_{\mathrm{Adv}}, \tag{S1}$$

where α is the weight of the adversarial contribution and we set it as $\alpha = 0.001$ in this work. The adversarial loss represents the (in)accuracy of the discriminator classification of the outputs of the Generator (into fake and true). For $\mathcal{L}_{\mathrm{Adv}}$, ref. [1] provides the detailed definition. It is considered that the information can be extracted most efficiently from the Discriminator when the discriminator loss $\mathcal{L}_{\mathrm{D}}$ is around 0.5. In this work, to maintain the value of $\mathcal{L}_{\mathrm{D}}$ in the vicinity of 0.5, adaptive training is carried out. In this special training protocol, the training for the discriminator is repeated if $\mathcal{L}_{\mathrm{D}}$ is larger than 0.6 and that for the generator recurs if $\mathcal{L}_{\mathrm{D}}$ is less than 0.45. Although in many cases such adaptive loops are performed for each minibatch, in this work, the adaptive loops are over the whole dataset. We have employed these precise protocols because we found them to be better than other options, after trials and errors.

C. Normalization of the data

All the data used for the training are normalized so that most (not all: see below) resulting pixels are in the interval $[0, 1]$. This is simply done by applying the following formula:

$$\tilde{x} = (x - x_{\mathrm{lb}})/x_{\mathrm{scale}}, \tag{S2}$$

where x denotes the variable of interest (one of the temperature, precipitation, sea-level pressure, or topography), $\tilde{x}$ is the normalized value, and x_{lb} and x_{scale} are the lower bound and the scale of the variable x, respectively. The precise values of x_{lb} and x_{scale} for each variable are summarized in Table S2. Such normalization is known to allow the network to handle variables with different physical dimensions (in this study, temperature, precipitation, pressure, and altitude) in a unified manner and enhance training efficiency. We stress that for precipitation, this normalization does not guarantee that all the resulting values are less than unity since rare events exceed the threshold value of 100 mm/day (the maximum value in the test samples is 912 mm/day). We employed this normalization factor because this choice gave the best performance among the values that we investigated (1000, 100, and 10 [mm/day]).

TABLE S2. The parameters used for the data normalization

	x_{lb}	x_{scale}
Temperature	-50K	100K
Precipitation	0 mm/day	100 mm/day
Sea level pressure	950 hPa	1050 hPa
Topography	0 m	4000 m

D. Precise measurement protocol of KL divergence

Technically, D_{KL} tends to infinity when $P^{\mathrm{GT}}(x) \neq 0$ and $P^{SR}(x) = 0$ holds for a value x (or more technically speaking, for a bin including x), and vice versa. Problematically, this usually happens in real situations because of the limited number of samples: the probabilities of finding rare events are regarded as zero when the number of samples is finite even though they should be small but nonzero in the "true" distribution that is expected to be obtained when the sample number becomes infinite. This results in an undesired infinite value of D_{KL}. To avoid such a trivial artifact, we applied the Gaussian smoothing function $f(x) = \frac{1}{\sqrt{2\pi}w} \exp\left\{\frac{-(x)^2}{2w^2}\right\}$ as:

$$P(x) = \frac{1}{Z\Delta} \sum_{i}^{N} f(x - x_i), \tag{S3}$$

where w^2 is the variance of the Gaussian and determines the smoothing width, Z is the normalizing factor that guarantees $\int P(x)dx = 1$, Δ is the bin width, and N is the number of data. $P(x)$ gives the probability of finding a sample in the interval $[x - \frac{\Delta}{2}, x + \frac{\Delta}{2}]$ and x_i is the value of the sample i. We fixed this hyperparameter w^2 and Δ to be $w^2 = 16$ and $\Delta = 2$ to obtain the results presented in Fig. 3 and Table 2 in the main text and Table S4. We confirmed that a change in the value of w by a factor of 4 does not change the qualitative results.

E. Locations where the correlation coefficients are evaluated

If we calculate the correlations between all the 400×400 grid points, the calculation cost becomes very expensive. Therefore, we extracted only the grid points where the observation stations of the Automated Meteorological Data Acquisition System reside. There are $N_{\mathrm{OS}} = 630$ stations within the system boundary of our study.

F. Topographic information

The precise topographic information about the locations considered in Fig. 3 is summarized in Table S3. The pixels within the region specified by the min/max of the latitude/longitude compose each site. Since, in our case studies, a single pixel in the high-resolution data has a linear dimension of 0.025 degrees in terms of both latitude and longitude, the regions of sites in Table S3 are all composed of 100 pixels.

II. FURTHER DETAILS OF RESULTS

A. Precise values of statistical indicators

We summarize the precise values of D_{KL} and MSE_{Jan} for each site in Table S4.

TABLE S3. Precise information of the location of each site

	Latitude		Longitude	
	min	max	min	max
(**A**) Akita	39.475°N	39.725°N	140.1°E	140.35°E
(**B**) Tokyo	35.625°N	35.875°N	139.65°E	139.9°E
(**C**) Niigata	37.65°N	37.9°N	139.0°E	139.25°E
(**D**) Shizuoka	34.95°N	35.2°N	138.25°E	138.5°E
(**E**) Nagoya	34.8°N	35.05°N	136.875°E	137.125°E
(**F**) Kanazawa	36.4°N	36.65°N	136.625°E	136.875°E
(**G**) Osaka	34.375°N	34.625°N	135.425°E	135.675°E
(**H**) Takamatsu	34.075°N	34.325°N	134.0°E	134.25°E
(**I**) Kouchi	33.525°N	33.775°N	133.55°E	133.8°E
(**J**) Izumo	35.125°N	35.375°N	132.75°E	133.0°E
(**K**) Hiroshima	34.225°N	34.475°N	132.525°E	132.775°E
(**L**) Oita	32.975°N	33.225°N	131.6°E	131.85°E

TABLE S4. Values of KL divergence of PDFs and MSE of correlation coefficients

	D_{KL}				MSE_{Jan}			
	πSRGAN	SRGAN	ψSRGAN	CDFDM	πSRGAN	SRGAN	ψSRGAN	CDFDM
Akita	1.62×10^{-3}	3.62×10^{-3}	3.03×10^{-2}	$\mathbf{9.08 \times 10^{-4}}$	1.43×10^{-2}	$\mathbf{1.32 \times 10^{-2}}$	1.94×10^{-2}	4.43×10^{-2}
Tokyo	3.40×10^{-3}	5.06×10^{-3}	5.36×10^{-3}	$\mathbf{1.47 \times 10^{-3}}$	6.41×10^{-3}	$\mathbf{5.75 \times 10^{-3}}$	8.96×10^{-3}	3.62×10^{-2}
Niigata	8.17×10^{-3}	6.32×10^{-3}	2.01×10^{-2}	$\mathbf{1.35 \times 10^{-3}}$	1.75×10^{-2}	$\mathbf{1.51 \times 10^{-2}}$	3.20×10^{-2}	7.26×10^{-2}
Shizuoka	2.63×10^{-3}	5.43×10^{-3}	4.15×10^{-3}	$\mathbf{1.27 \times 10^{-3}}$	8.40×10^{-3}	$\mathbf{7.06 \times 10^{-3}}$	1.13×10^{-2}	3.18×10^{-2}
Nagoya	2.32×10^{-3}	4.28×10^{-3}	8.72×10^{-3}	$\mathbf{1.65 \times 10^{-3}}$	$\mathbf{8.48 \times 10^{-3}}$	1.11×10^{-2}	1.61×10^{-2}	4.07×10^{-2}
Kanazawa	5.24×10^{-3}	4.24×10^{-3}	2.19×10^{-2}	$\mathbf{1.46 \times 10^{-3}}$	$\mathbf{2.67 \times 10^{-2}}$	3.91×10^{-2}	4.35×10^{-2}	8.77×10^{-2}
Osaka	2.06×10^{-3}	3.79×10^{-3}	1.68×10^{-2}	$\mathbf{1.51 \times 10^{-3}}$	1.39×10^{-2}	$\mathbf{1.25 \times 10^{-2}}$	1.61×10^{-2}	4.55×10^{-2}
Takamatsu	2.12×10^{-3}	2.65×10^{-3}	1.63×10^{-2}	$\mathbf{1.35 \times 10^{-3}}$	9.11×10^{-2}	$\mathbf{8.67 \times 10^{-3}}$	1.91×10^{-2}	4.41×10^{-2}
Kouchi	2.73×10^{-3}	8.29×10^{-3}	1.07×10^{-2}	$\mathbf{1.19 \times 10^{-3}}$	9.23×10^{-3}	$\mathbf{5.95 \times 10^{-3}}$	9.74×10^{-3}	2.62×10^{-2}
Izumo	1.20×10^{-3}	2.36×10^{-3}	3.81×10^{-3}	$\mathbf{9.62 \times 10^{-4}}$	$\mathbf{1.28 \times 10^{-2}}$	1.41×10^{-2}	2.98×10^{-2}	3.94×10^{-2}
Hiroshima	$\mathbf{1.80 \times 10^{-3}}$	3.31×10^{-3}	4.87×10^{-3}	2.01×10^{-3}	7.69×10^{-3}	$\mathbf{7.59 \times 10^{-3}}$	1.81×10^{-2}	2.35×10^{-2}
Oita	3.43×10^{-3}	3.23×10^{-3}	1.09×10^{-2}	$\mathbf{2.88 \times 10^{-3}}$	$\mathbf{1.01 \times 10^{-2}}$	1.19×10^{-2}	2.08×10^{-2}	3.05×10^{-2}
Average	3.06×10^{-3}	4.38×10^{-3}	1.28×10^{-2}	$\mathbf{1.50 \times 10^{-3}}$	$\mathbf{1.20 \times 10^{-2}}$	1.27×10^{-2}	2.04×10^{-2}	4.35×10^{-2}

(the best values are shown in bold letters for each row, each indicator)

B. Standard statistical indicators for single-image super-resolution

In this subsection, we present customary statistical measures that are widely employed in the domain of image processing. The first indicator is the mean squared error (MSE) of the pixel-based results $\mathrm{MSE}_{\mathrm{pixel}}$ defined as:

$$\Delta_k \equiv \frac{1}{N_l} \sum_l (p_k^{\mathrm{GT}}(l) - p_k^{\mathrm{DS}}(l))^2 \tag{S4}$$

$$\mathrm{MSE}_{\mathrm{pixel}} \equiv \frac{1}{N_{\mathrm{test}}} \sum_k^{N_{\mathrm{test}}} \Delta_k \tag{S5}$$

where Δ_k is the mean squared error of the kth sample and N_l is the total number of pixels that represent the locations on land. The remaining variables adhere to the definitions introduced in the main text. Following the discussion in the main text, we consider only the precipitation here.

Using this pixel-wise measure $\mathrm{MSE}_{\mathrm{pixel}}$, the peak signal-to-noise ratio (PSNR), which is usually used as a quantitative measure of the performance of single-image super-resolution tasks, can be defined as:

$$\mathrm{PSNR}_P = 10 \cdot \log_{10} \frac{P^2}{\mathrm{MSE}_{\mathrm{pixel}}} \tag{S6}$$

where P stands for the maximum signal intensity. In the case of image processing tasks, P is trivially determined by the possible maximum intensity, e.g., it should be 255 if the pixel is expressed by 8-bit information. However, in the current situation, the peak signal intensity is not known in advance and thus we employed the maximum values observed in the test samples. There are "two different peak signals": the one observed in the downscaling results and the one in the ground truth. In Table S5, we compare the values of PSNR that are measured using the peak signal of the ground truth $P_{\mathrm{GT}} = 9.12$ and the ones in the downscaling results P_{DS}. Despite a very large scale factor of fiftyfold, the results for all methods are remarkably favorable when compared to the benchmarks established in the image processing field. This is simply because the precipitation field exhibits very small values in most sites in most samples compared to the peak signal, and so are the errors.

TABLE S5. MSE, PSNR, and P_{DS} of machine learning-based methods

	$\mathrm{MSE}_{\mathrm{pixel}}$	$\mathrm{PSNR}_{P_{\mathrm{GT}}}$	$\mathrm{PSNR}_{P_{\mathrm{DS}}}$	P_{DS}
πSRGAN	7.99×10^{-3}	40.2	39.1	8.10
SRGAN	8.35×10^{-3}	40.0	37.6	6.90
ψSRGAN	1.15×10^{-2}	38.6	36.4	7.10

To offer a more intuitive understanding of the difference in the accuracy of each approach (πSRGAN, SRGAN, ψSRGAN, and CDFDM), we have included Fig. S2, which illustrates the difference plots for the single-sample visualization (corresponding to Fig. 2 in the main text), and Fig. S3, which depicts the spatial distribution of the correlation coefficient (Fig. 4 in the main text). These figures substantiate the assertions made in the main text.

In Fig. S2 (A), the disparities between the temperature fields of the ground truth and those generated by the downscaling techniques are illustrated. Interestingly, the magnitude of the error is minimal in CDFDM and maximal in ψSRGAN in accordance with the statistical metrics of precipitation. However, since ψSRGAN incorporates the LR information about the temperature field like other SRGAN-based methods, this inferior performance for the temperature field is unexpected. We need to conduct a comprehensive statistical analysis to draw a definitive conclusion: such analysis falls outside the scope of this paper.

Fig. S2 (B) shows the differences in the precipitation field between the ground truth and the downscaling results. Here, while large errors are relatively widely distributed in the cases of ψSRGAN and CDFDM, only small and localized errors are seen in the cases of πSRGAN and SRGAN.

In Fig. S3, the errors in the spatial distribution of the correlation coefficient (again, defined as the simple root mean squared errors from the ground truth) are displayed. Notably, in the instances of CDFDM, we note extensive regions exhibiting dark colors, which denote significant discrepancies. Specifically, with respect to CDFDM, the areas of dark color align with prominent ridgelines. This is indicative of the spatial layout of the correlations, originating from the topography, being inadequately represented by CDFDM. On the other hand, in the columns of SRGAN-based methods, again even including ψSRGAN, we observe light hues across nearly all locations. This outcome serves to demonstrate the exceptional precision of SRGAN-based methods and, in particular, our approach πSRGAN.

III. GENERALIZATION ABILITY TEST

In this section, we present the outcomes of the generalization capability test of SRGAN. Specifically, we performed downscaling computations on the test samples covering the region spanning from 135.625 to 145.625 degrees east and from 35.625 to 45.625 degrees north, utilizing the Generator network featured in the main text. The network was initially trained on the area illustrated in Fig. 3M in the main text, ranging from 130.625 to 140.625 degrees east and 30.625 to 40.625 degrees north. Thus, the samples employed in this generalization test were shifted by five degrees both in the north and east directions, relative to those utilized in the original analysis. We have calculated the simple probability distribution function of the precipitation $P(p)$ and D_{KL} between $P^{\mathrm{GT}}(p)$ and $P^{\mathrm{SRGAN}}(p)$ (the information

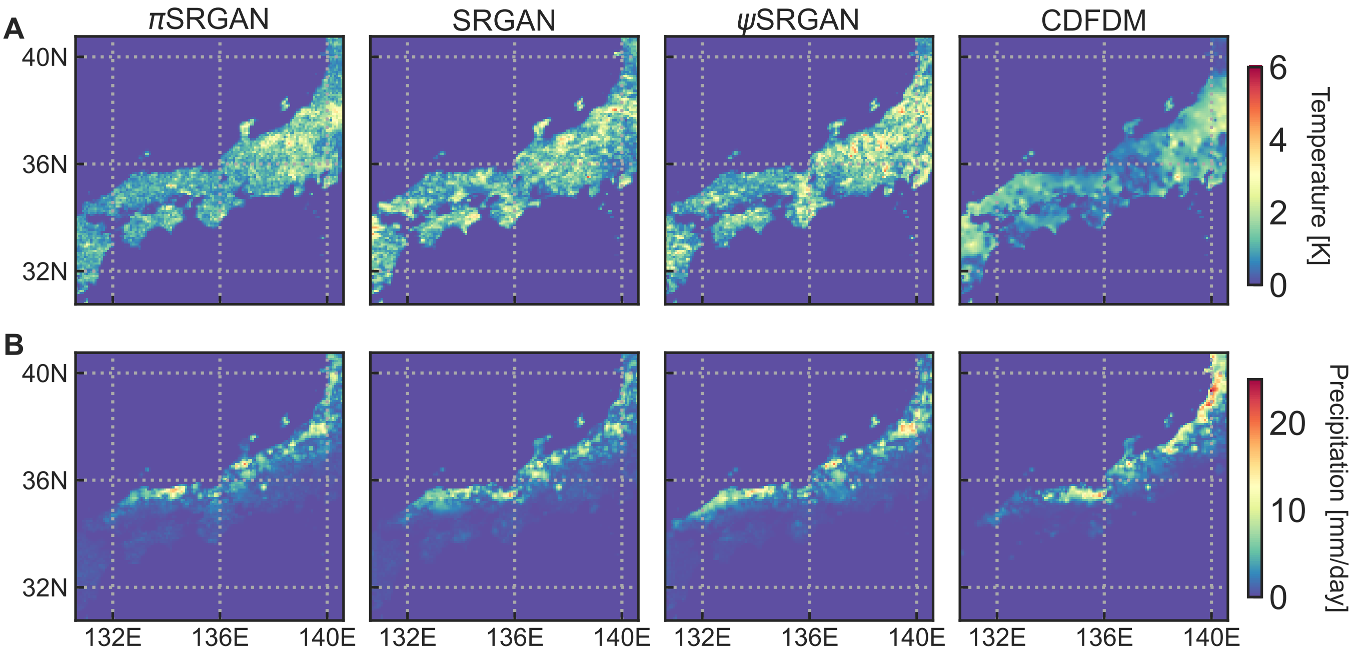

FIG. S2. Difference plots for single-sample visualization. Distributions of errors in (A) temperature and (B) precipitation obtained from πSRGAN, SRGAN, and CDFDM are compared. The data from January 24, 2008, are displayed, as in Fig. 2 in the main text.

presented in Fig. 3) for 8 representative locations depicted in Fig. S4 (I). Notice that two out of these eight sites are the same ones considered in Fig. 3 in the main text (Niigata and Kanazawa). The results of $P(p)$ are shown in Fig. S4 (A-H) and $D_{\rm KL}$ for each site is shown in Fig. S4(J). We note that, unlike Fig. 3(P) in the main text, we needed to employ the logarithmic scale for the ordinate of Fig. S4(J) because the accuracy becomes worse by orders of magnitude for several sites (e.g., Akita and Kanazawa). These results indicate that the precision of our method deteriorates significantly when we alter the target location for the downscaling (quantitatively speaking, the value of the KL divergence becomes more than ten times higher). Errors are observed not only in the high precipitation regime, where $P^{\rm GT}(p)$ attains very small values, but also in the low precipitation regime, e.g., in Fig. S4(B,E,G,H). The absence of generalization capacity is an anticipated characteristic as our network has been optimized for a specific geographical region.

[1] K. Stengel, A. Glaws, D. Hettinger, and R. N. King, Adversarial super-resolution of climatological wind and solar data, Proceedings of the National Academy of Sciences **117**, 16805 (2020).

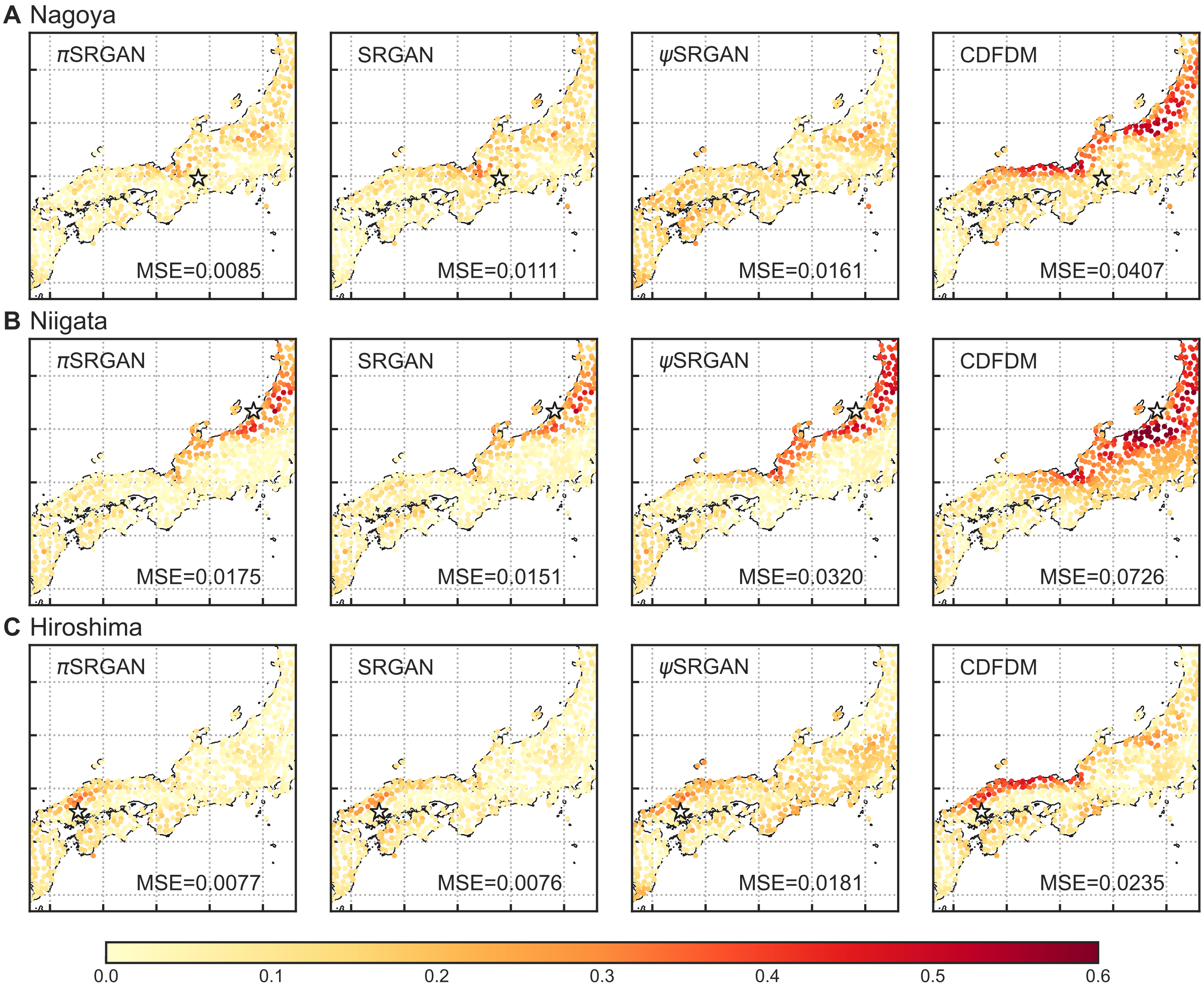

FIG. S3. Spatial distribution of the mean squared error in the correlation coefficient for precipitation. The results of January obtained with the πSRGAN, SRGAN and CDFDM are compared in the case of the reference point of correlation at Nagoya [35.1667°N, 136.965°E] (A), Niigata [37.9133°N, 139.0483°E] (B), and Hiroshima [34.365°N, 132.4333°E] (C). The dot color indicates the values of $|C^{GT}_{\text{Jan}}(l,l') - C^{DS}_{\text{Jan}}(l,l')|$ between the location of the dots and the reference site. The reference points are represented by star symbols.

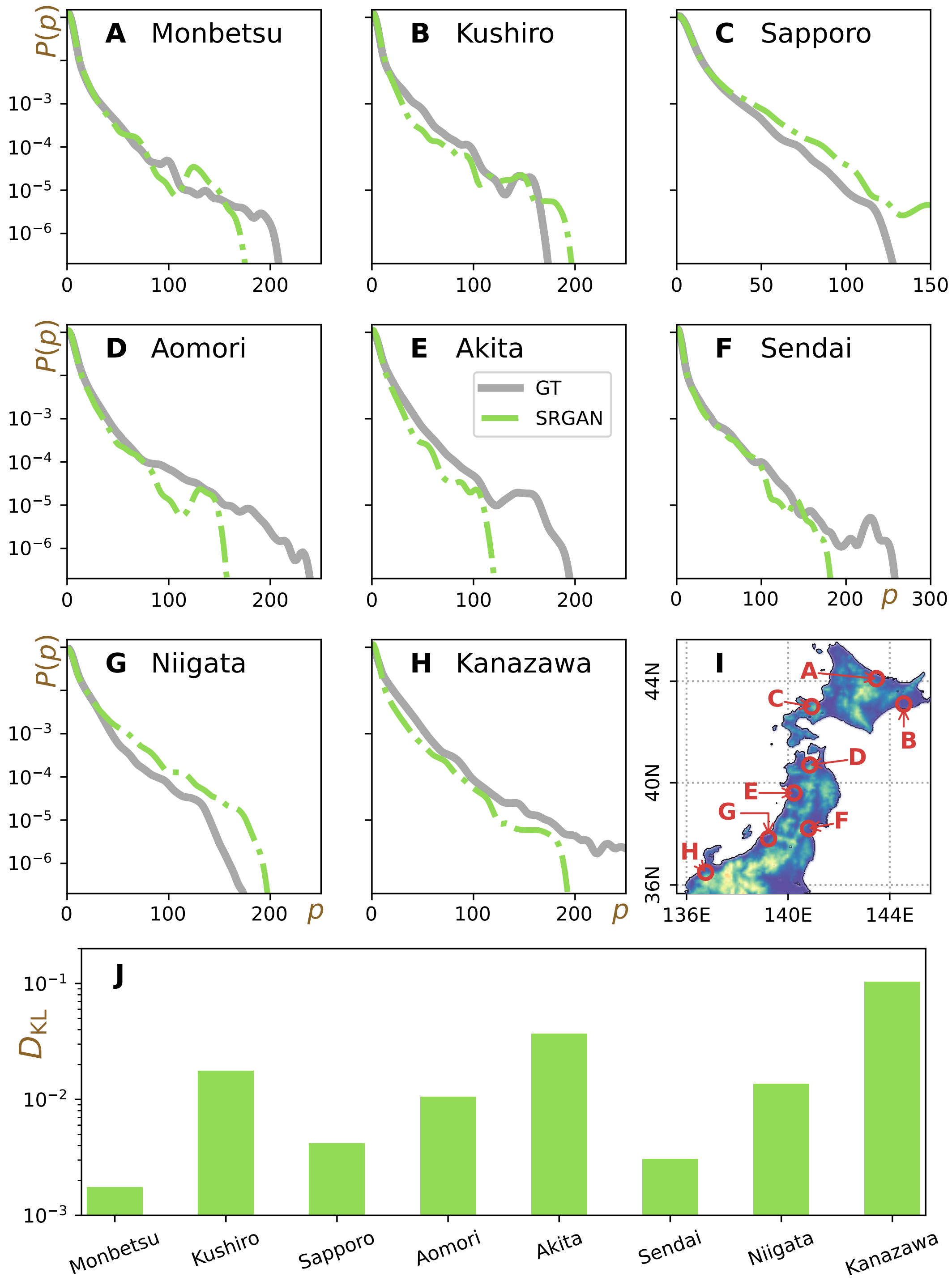

FIG. S4. Results of generalization ability test: statistics of precipitation calculated for a region different from ones used for training. (A-H) The probability distribution functions (PDFs) $P(p)$ as a function of the precipitation p at each site. 8 representative sites are chosen from the entire computational domain of the test. The different ranges of the abscissa are employed for different sites. (I) The normalized topographic information and the locations of 8 sites of panels (A-H). (J) Bar plot of the Kullback-Leibler divergence D_{KL}.
